# The Psychology of Sports Injuries in Children and Adolescents: Psychosocial, Developmental, and Recovery Aspects to Injury

**DOI:** 10.3390/ijerph22101509

**Published:** 2025-09-30

**Authors:** Linh-Nhu Hoang, Pradnya Joshi, Dilip R. Patel, Roger W. Apple

**Affiliations:** 1 Department of Psychology, Western Michigan University, Kalamazoo, MI 49008, USA; nhu.hoang@wmed.edu; 2 Department of Pediatric and Adolescent Medicine, Western Michigan University Homer Stryker M.D. School of Medicine, Kalamazoo, MI 49008, USA; pradnya.joshi@wmed.edu (P.J.); dilip.patel@wmed.edu (D.R.P.)

**Keywords:** sports injury, psychosocial factors and stressors, psychosocial responses, children and adolescents, youth, developmental aspects, rehabilitation, behavioral health interventions

## Abstract

Participation in sports and the presence of sports injuries have a lasting impact on youth athletes’ physical, cognitive, and emotional development and sense of self-identity. There is an ongoing growth in participation in sports for youth, as well as growing literature on the epidemiology and outcomes of sports-related injuries. However, there is a paucity of published research regarding the psychological aspects of sports injury, including psychosocial factors, stressors, and responses, from the perspective of young athletes. Key risk factors include the youth’s sex, the types of sports activity, and any previous injuries. Psychosocial models, such as the stress-injury model, help explore such risk factors and their relationship to outcomes of stress. Implications for sports injury outcomes vary within the pediatric population, and the recovery and rehabilitation process requires integrated healthcare to optimize health and mental health outcomes. This review aims to describe the psychosocial factors related to sports injuries in children and adolescents, provide an understanding of sports injury models for youth athletes, and point to recovery and prevention through integrated behavioral health interventions. Based on a literature search, we identified 40 articles most relevant to our aims to explore psychosocial factors and stressors, predisposing and risk factors, and developmental aspects of sports injuries in children and adolescents.

## 1. Introduction

Participation in sports and recreational activities for children and adolescents is positively correlated with the physiological and psychological benefits. Youths who engage in sports are likely to have an increased level of physical activity later in life, while increasing their level of knowledge of nutrition, exercise, and overall health [1]. The youth developmental period is critical for cognitive, emotional, and motor enhancements; therefore, it is important to consider brain and physical development as it pertains to sports participation during this period. Sports participation contributes to a variety of health-related benefits such as muscle mass, cardiopulmonary endurance, increased endorphin levels, increased neurotransmitter production, and attenuation of the hypothalamic–pituitary–adrenal (HPA) axis response to stress [1]. While there are clear health benefits for youth development upon engagement in sports or activities, this engagement offers child and adolescent growth opportunities, leading to enhanced self-esteem and stress reduction [1]. Participating in teams and building sportsmanship skills allows youths to build self-confidence and a sense of responsibility. Sports participation is also essential for establishing autonomy during youth self-identity development. Specifically, for adolescents, sports engagement can help achieve a greater sense of athletic and self-identity, ultimately leading to a stronger sense of self-actualization [1,2]. Athletes with high athletic identity generally have higher self-confidence, discipline, and social interactions. Theories related to athletic identity posit that it can occur in the spectrum of a small part of one’s identity to a larger, all-encompassing part of another’s life and that identity affirmation and inclusion are achieved through active participation in sports and activities [1]. Injuries are inevitable consequences of sports. Participation in sports and recreational activities may lead to the risk of injury that can threaten short- and long-term physical and mental health.

While there has been an exponential growth in participation in sports and recreational activities for younger children and adolescents, there is a growth in the implications of mental health problems, which recognize the importance of psychological well-being in the adolescent and pediatric population [2]. Sports-related injuries appear to have a bidirectional relationship with the presence of mental health concerns, as athletes experience similar, if not higher, rates of mental health conditions than nonathletes [3,4]. Sports injury has been identified as a sport-specific risk factor for poor mental health. However, for many cases of higher rates of mental health conditions, athletes are seeking mental health treatment at lower rates than nonathletes. However, little is known about the mental health of children and adolescent athletes [5]. The literature on sports-related injuries for children and adolescents is far behind the literature on sports-related injuries in adults or collegiate athletes. This article aims to discuss sports-related injuries in children and adolescents with regard to psychosocial factors, recovery, and prevention.

### Sports Injury as Perceived by Athletes and Trainers

When there is an emphasis on youth’s level of responsibility for their athletic role, there is a higher tendency for their self-esteem, motivation, and outlook on sports to be based on their level of competence, performance, and achievement [1]. This form of pressure may set a precedent that youth athletes must always perform at their best, even if it poses a greater risk of injury. The most common outcomes of injury in children and adolescents include general pain, immobility, disability, time lost from sports or activities, absence from school, and other developmental issues [1]. These outcomes may ultimately lead to difficulty in coping with the injury, reduced self-confidence, and feelings of hopelessness among youth. Required rest from sports often results in boredom for youth, which is associated with increased stress and loss of social contact [1]. Adolescent athletes with a history of disordered eating, in particular, struggle with time loss from sports or activities due to their concerns about body image and eating. Additionally, elite youth athletes tended to exhibit a higher prevalence of eating disorder symptoms than non-elite athletes. Studies have found that, in some youth athletes, emotional trauma was experienced following injury for those who strongly identified as athletes than for those who did not strongly identify as athletes [1].

A study of injured athletes reported an underuse of motivation and adherence to strategies for recovery in the athletic training room [6]. There is a lack of education in this area for athletic trainers (ATs), and athletes have reported a lack of motivation in some ATs. Stigma around mental health as it pertains to sports-related injuries may also exist both inside and outside the sports environment [3]. This influences athletes’ willingness to engage in mental health treatment as they may have a concern about playing time if their mental health condition is known or believe in the notion that having a mental health condition is considered a “weakness.”

## 2. Materials and Methods

### 2.1. Protocol

The protocol was established as a narrative review with systematic elements of the guidelines outlined in the PRISMA Search Strategy. The result of not using the full PRISMA framework was the increased risk that other relevant literature was missed during the search. Further input and subsequent changes were made based on the discussions within the research team.

### 2.2. Search

A literature search was conducted in October and November 2024 to explore psychosocial factors and stressors, predisposing and risk factors, and developmental aspects of sports injuries in the pediatric population. Information sources for this review included PubMed, PsycINFO, Scopus, and Embase databases, with data limited to English-language studies published from 2014 to 2024. These databases were identified by the WMed Librarian team, which included relevant articles to the key search topics. This timeframe was chosen to highlight the contemporary findings on psychosocial factors and stressors related to sports injuries in children and adolescents.

The initial search strategy utilized the terms “sports injur*”, “sports-related injur*”, “athletic injur*”, “athletic train*”, “child*”, “adolescen*”, “youth,” “teen,” “pediatric*”, “developmental,” “psychosocial factor*”, “psychosocial response*”, “stress*”, “risk factor*”, “predispos*”, “developmental aspect*”, “rehabilitation,” “medical professional*”, “intervention*”, and “behavioral health.”

### 2.3. Inclusion and Exclusion Criteria

To be included, articles needed to address psychosocial factors and stressors, predisposing and risk factors, and developmental aspects in the context of sports-related injuries within the pediatric and adolescent populations. Peer-reviewed observational studies, longitudinal cohort studies, systematic reviews, and meta-analyses were also included. Case reports, comments, or letters to the editor were excluded. Articles were also excluded if the participants were older than 18 years old. Forty articles were included in this review.

## 3. Results

**Figure 1:**
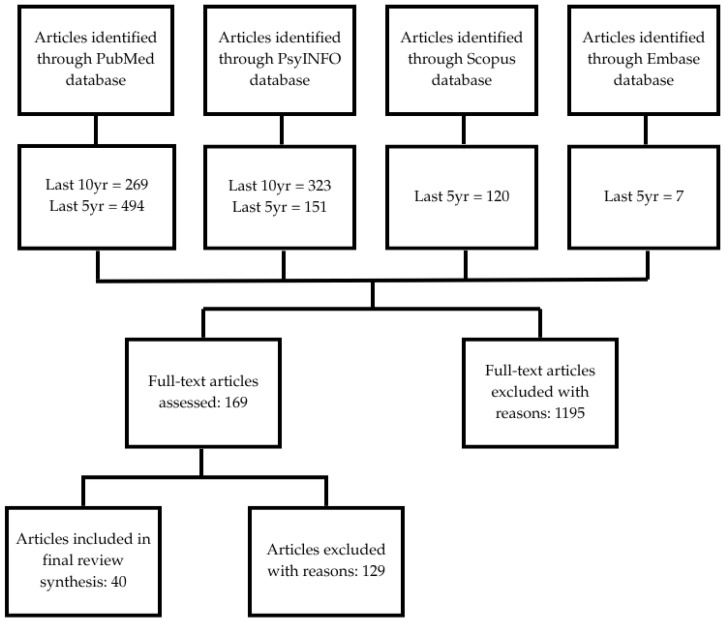
Flow diagram of articles assesses for eligibility. A total of 169 full-text articles were identified through database searches and screened for review. After screening and application of eligibility criteria, 40 articles were included in the final review.

PubMed searches yielded 269 results in the last 10 years and 494 results in the last 5 years. A similar search of PsycINFO yielded 323 results from the last 10 years and 151 results from the last five years. A similar search using Scopus yielded 120 results. The Embase database included seven Embase-only articles. Forty articles were included in this narrative review (Figure 1).

### 3.1. Developmental Aspects

Participation in sports in young adolescent and pediatric populations has long been associated with significant benefits for young athletes [7]. However, these activities result in a weighted risk of injuries, which can ultimately lead to short- and long-term complications [7]. Certain sports injuries may lead to developmental complications in the youth. Sports participation and risk for injuries vary in growth and development that are dependent on gender and age group factors, as well as whether the youth have physical, cognitive, behavioral, or neurodevelopmental disabilities [8]. Concussions related to sports are an important consideration for young adolescents who participate in nearly all sports at all training levels [9]. It has been shown that concussions have resulted in cognitive issues, with one study showing that females with two or more prior concussions have worse verbal or visual memories than those with zero prior concussions [10]. Given the risk of concussion, there are effective measures to protect against such injuries. This is crucial because early intervention and preventative measures can decrease the likelihood of severe or long-term damage. These protective strategies for children and adolescents emphasize the importance of personal protective equipment, the implementation of policy and rule changes, tailored training programs, and comprehensive management approaches aimed at reducing the risk of recurrent concussions [11].

Beyond the physical symptoms, injuries in youth athletes can lead to significant psychological effects as well [7]. One study found that some athletes under the age of 21 have been found to exhibit symptoms of post-traumatic stress disorder (PTSD) following an anterior cruciate ligament (ACL) rupture, with trauma being more pronounced among those aged 15–21 years compared to younger athletes under the age of 14 [2,7,12]. Research has been conducted on the incidence of mental health outcomes such as depression, anxiety, post-traumatic stress, and high-risk behaviors among adolescent athletes following injuries [7,13]. This highlights the need for further research to better understand and address the mental health challenges faced by injured youth athletes. Expanding the current area can help protect young athletes from developing serious mental health conditions that can impact their development and ensure timely support and intervention.

### 3.2. Predisposing and Risk Factors for Injuries

Research has shown that certain demographics and groups are at a higher risk of sports-related injuries. These risk factors are important to consider in the pediatric population, as injuries in adolescents can cause an arrest in growth and development [2,14]. Factors such as sex, age, type of sport, and prior injury history are a few elements that are explored in the literature as risk factors for injuries sustained in the pediatric and adolescent populations; however, data are limited on the psychological and psychosocial risk factors for sport-related injuries in children and adolescents.

#### 3.2.1. Sex

Sex is one component cited in the literature as a potential risk factor and should be considered when assessing risk in pediatric and adolescent populations. In a study of 4418 youth ice hockey players in Canada, it was found that sex was one of the greatest factors contributing to injury [15]. Female players reported a 90% greater rate of injuries during practice than male players. Additionally, those who participated in “female-only” leagues experienced greater rates of injuries than those who participated in co-ed leagues [15]. Al-Qahtani and colleagues found similar results in their studies, also noting that females are often at higher risk of injury [16]. This risk factor is not an isolated finding in hockey, as similar trends have been found in basketball and floorball athletes [17].

Males and females have also been shown to have different risk rates for specific injuries. While females were found to be more susceptible to knee injuries, foot or ankle injuries, bone stress injuries, and concussions, males were found to be more susceptible to hip, groin, and hamstring injuries [18]. Being aware of risks may help in taking proper precautions to minimize incidents of injury. Implementing sport-specific guidelines and strategies can also be a step toward minimizing injuries in both male and female athletes. These strategies could include proper training techniques, adequate rest periods, and the use of protective equipment tailored to the type of sport.

#### 3.2.2. Type of Sport Activity

The type of sports played by children and adolescents can be an important determinant in evaluating the risk of injury. Different sports involve distinct physical movements and specific motions that can contribute to particular types of injuries more commonly observed in sports. These repetitive and sport-specific actions, such as throwing in baseball, jumping in basketball, or tackling in football, can place stress on targeted areas of the body, which, in turn, can increase the likelihood of sport-specific injuries. In a 2000 study, 677 charts of children and adolescents were retrospectively reviewed, and of those charts, six sports were identified as comprising more than 4% of the cases. These sports included basketball (19.5%), football (17.1%), baseball/softball (14.9%), soccer (14.2%), rollerblading (5.7%), and hockey (4.6%) [19]. The mechanisms of these injuries also vary greatly depending on the sport. In the same study, contact with a person or object accounted for 87% of injuries in baseball or softball as opposed to only 39% in basketball and an even lower 8% in rollerblading [19]. Contact with the ground instead made up 89% of injuries in rollerblading, while only 19% in basketball, and an even lower 3% in baseball or softball [19]. Looking at the aggregated data not separated by sport would show that 50% of overall injuries were caused by contact with a person or object, so it is important to consider the type of sport when targeting injury prevention, since effectiveness can differ depending on the sport being considered. It has been shown that it is possible to minimize specific injury types with proper precautions and equipment. For example, studies have shown that the occurrence of mouth and face injuries in football has decreased owing to protective equipment usage [19].

The aforementioned results focused on individual sports statistics, but in reality, adolescents may train in multiple sports over the course of their lives. Having a variety of sports to train has been shown to be beneficial in reducing the likelihood of overuse injuries, as those who have an early specialization in one sport are more likely to develop neuromuscular deficits that are a primary cause of acute and chronic injuries [20].

#### 3.2.3. Previous Conditions and Injuries

Other factors associated with increased injury rates include age (older age groups experiencing higher incidences of injury), history of concussions, and previous injuries sustained within the past 12 months [15]. Interestingly, neither the year of play nor the level of play was a significant risk factor for injuries. However, it is documented in the literature that some studies have reported mixed results on these variables [15]. For example, a study examining biomechanical risk factors among female adolescent volleyball players suggested that targeted injury prevention programs could help address factors such as knee valgus, which has been associated with higher Beighton scores (a measure of joint hypermobility) [21]. As greater Beighton scores were identified as a risk factor for increased knee valgus, the study also recommended incorporating targeted neuromuscular training to complement injury prevention programs and further mitigate this risk [21]. Awareness and understanding of these risk factors in the pediatric population can help drive targeted injury prevention and education for young athletes.

### 3.3. Psychosocial Factors and Stressors

Much of the literature related to sports injuries in children and adolescents has focused on the physical implications of injuries while neglecting the psychosocial impact as it pertains to the mind–body connection for injury in youth [1]. Sports-related injuries for children and adolescents can have a lasting negative impact on their cognitive, emotional, and social lives. When athletes are faced with a variety of highly stressful demands, these stressors can significantly negatively affect their responses to injury [22]. The influence of psychosocial behavioral factors on the occurrence of injuries in sports and recreational activities has received increased attention [23]. An athlete’s cognitive appraisal of their sports injury, as well as their emotional and behavioral responses, are influenced by a range of personal, situational, and environmental factors [4,24]. The psychosocial factor that is most closely related to emotional changes, particularly sports-related injury, is Bandura’s self-efficacy theory [25]. This factor is concerned with personal competence and posits that an individual’s level and strength of experiencing an inner belief and competence in learning or performing a task or activity may be successful, regardless of their history of events. Thus, self-efficacy beliefs have a positive impact on the performance of various athletes, regardless of their age or ability. The impact of sports-related injuries on youth can change their lives and increase their vulnerability to mental health conditions, such as depression and post-traumatic stress disorder. Psychosocial impacts of injury include time loss, fear or occurrence of re-injury, coping difficulties, reduced confidence, and feelings of helplessness [1]. Some young athletes recovering from an injury may also experience a loss of identity and a significant decrease in their quality of life. Athletic identity serves as both a strength and vulnerability factor for young athletes’ sports injury processes and outcomes. A narrative review of athletic identity and sports injury processes and outcomes in youth athletes found that high levels of athletic identity were associated with various factors, including reluctance to report injuries, willingness to play through pain, and intensified physical and psychological symptoms [26]. Previous research has failed to account for variables that interact with life stressors that influence sports-related injury risk. However, research has suggested that high-stress athletes are more likely to incur injury than their low-stress counterparts [23].

### 3.4. Models of Sports Injury and Mental Health

Models related to sports injuries have been studied and designed to contribute to the understanding of the relationship between injuries and mental health outcomes. Applying a multivariate model of sports injury etiology can lead to greater success in predicting sports injuries and help guide effective prevention strategies during the treatment and rehabilitation process.

#### 3.4.1. Stress-Injury Model

The broad idea of the stress-injury model is that when athletes experience sports-related stressful situations, such as injury, their history of stressors, personality characteristics, and coping resources contribute interactively or in isolation to the stress response [27]. Thus, the stress-injury model hypothesizes that individuals with a history of sports-related stressors, personality characteristics that exacerbate the stress response, and very few coping resources will exhibit greater physiological activation and attentional disruptions than individuals with the opposite psychosocial model. The center of the model, then, is a bidirectional relationship between the individual’s cognitive appraisal(s) of a potentially stressful situation and the physiological and attentional outcomes of stress [27]. For example, athletes appraise the demands of stressful situations, such as the immediate outcome of an injury, adequacy of the athlete to meet those demands, and potential consequences of failure or success. As a result, the stress response is more likely to activate and manifest itself physiologically, attentionally, and in the perception of higher state anxiety when the athlete perceives that it is important to succeed.

#### 3.4.2. Integrated Model of Psychological Response to the Sport Injury and Rehabilitation Process

Psychological consequences, such as cognitive, emotional, and behavioral responses, occur following sports injury. Several conceptual models provide a reference framework for understanding the psychological responses associated with sports injuries. As a result, an integrated model of psychological responses to sports injuries and rehabilitation processes has been developed [28]. The integrated model then includes personal and situational factors, behavioral and emotional responses, and cognitive appraisal while considering the grounded theory approach and process-based models. The model recognizes the interaction between athletes’ cognitive appraisal of their sport injury and their emotional and behavioral responses as a bidirectional and dynamic cyclic process [24].

While both the above-mentioned models can be relevant to both child and adolescent populations, it is important to distinguish the focus of the two. The stress-injury model highlights the bidirectional relationship between stress and health outcomes. The Integrated Model of Psychological Response to the Sport Injury and Rehabilitation Process, on the other hand, further expands on the stress-injury model to highlight the journey of injury and rehabilitation for the athlete.

### 3.5. Recovery

Sports injury recovery and athletic performance are highly linked, with the recovery process having an important effect on athletes’ ability to perform at their highest level. Psychological resilience plays a crucial role in the athletic injury recovery process and performance [29]. Psychological resilience helps athletes maintain motivation and adhere to rehabilitation programs for recovery. Athletes with high resilience are generally more likely to persevere in conflicts and stick to their recovery plan, as they might derive meaning and purpose in the rehabilitation process. Psychological resilience provides an optimistic attitude linked to increased self-confidence, self-efficacy, belief in one’s capacity to overcome barriers, and better physical recovery results [29]. However, the existing literature on the relationship between psychological resilience and injury recovery focuses primarily on the adult population. There is little evidence on the impacts of psychological resilience in the pediatric population. Thus, future research should further examine the relationship between high psychological resilience and levels of motivation and recovery for young athletes.

#### Psychosocial Responses During Rehabilitation

Psychosocial responses to injury include cognitive appraisals, emotional responses, and behavioral responses, which vary across different phases of the rehabilitation process. The three physiological phases that have been traditionally conceptualized in rehabilitation programs are acute injury, repair, and remodeling [30]. These phases have been used as blueprints for guiding treatment, and although they have been proven effective, athletes’ psychosocial responses have not been explicitly considered during the rehabilitation process. Researchers have proposed a shift in using an integrated model of response to the sports injury and rehabilitation process, which can provide a theoretical framework for the long-standing phased approach [30]. In this integrated model, athletes can respond to their injuries in a variety of ways, and the number of preinjury factors, such as biological, physical, psychological, and sociocultural stressors, can influence both injury occurrence and subsequent reactions to injury [30,31].

Psychological responses, in addition to emotional and behavioral responses to sports injuries, have also been studied. Psychological responses include feeling devastated, which is reflected by feelings of shock and emptiness, and feeling dispirited, which is reflected by feelings of apathy, lack of motivation, and frustration [22,32]. Resilience to stress has been found to help mitigate psychological responses to injury. Researchers found that adolescents with higher “mental toughness” and resilience coped with stress more effectively and reported lower levels of symptoms related to depression, anxiety, and obsessive-compulsive disorder [31]. This emphasizes the importance of identifying the stressors that apply to specific athletes and the consideration of stress-reduction techniques to be implemented during treatment.

Athletes may experience a new onset or an exacerbation of mental health symptoms throughout the rehabilitation process [3]. Thus, identifying and addressing the psychosocial factors impacting an athlete’s readiness to return to their sport can help improve rehabilitation outcomes.

### 3.6. Role of Training and Conditioning

The process of re-training and reconditioning injured athletes during their injury-rehabilitation process is crucial to the success of their return to sports or the general recovery period. Utilizing a coordinated team approach during this injury-rehabilitation process requires medical professionals and other healthcare practitioners to provide appropriate information that addresses the athlete’s specific needs to maximize physical and mental health outcomes [33,34]. Successful outcomes in the care and prevention of athletic injuries involve the involvement of a multidisciplinary team.

### 3.7. Medical Professionals’ Roles in Sports Injury Rehabilitation

Musculoskeletal injuries are the most common reason for primary care visits in the adolescent and pediatric population [35]. Athletes’ help-seeking behaviors from professionals during the injury-rehabilitation process vary and are complex and contingent on their phase in the process [36]. Athletes perceive the role of a sports medicine professional (SMP) as important in attending to the physical outcome of the injury [32]. Their role is especially important to athletes as they tend to be the ones to first attend to the injury and are often present immediately after the injury has taken place [32]. While SMPs are adequately trained to address the physical aspects of injury, many report feeling inadequately trained to address the psychosocial aspects of injury. Education provided by coaches, trainers, or professionals has often been decontextualized and fragmented [33]. Thus, it is ideal for athletes to have access to a wide range of allied health professionals during their injury rehabilitation process, utilizing a comprehensive approach to cover all aspects of injury, including physical, psychosocial, and behavioral.

Following medical guidance, participation in rehabilitation exercises, attending physical therapy sessions, and maintaining physical activities as directed by healthcare specialists are all essential components of the sports injury recovery process [29]. It is important for medical providers and other healthcare professionals to understand how mental health concerns manifest in athletes and how to help them navigate potential barriers to treatment [3,37]. During the period in which young athletes return to play, where the body must adapt to the demands of the sport while reducing the probability of re-injury, the assistance of medical or healthcare professionals in addition to the youth’s coaches is critical for ensuring a safe and effective transition [29]. This includes monitoring young athletes’ bodies and consistently implementing injury prevention techniques, which are discussed in the next section. Such monitoring involves regular checkups with medical providers or other healthcare professionals who can help identify and address any underlying concerns. Coaches and parents may also play a key role in having the ability reducing the risk of injury by providing the right equipment and gear, promoting safe play conditions, and ensuring adequate supervision [37].

## 4. Discussion

The prevention of sports-related injuries requires ongoing assessment and treatment. A team-oriented approach for prevention from a multidisciplinary team provides an effective outcome for athletes [34,38]. The first step in ongoing assessment is to monitor the overall health of elite youth athletes over extended periods of time in various training and competition settings [5]. An ongoing treatment approach involves the integration or culmination of a variety of interventions, such as educational interventions, behavioral health interventions, psychoeducation, skills training, and maintenance tools.

### 4.1. Educational Intervention in Psychology of Injury

Seeking psychosocial interventions is one of the 12 main content areas in several athletic training education programs [6]. The goal of an educational intervention in the psychology of injury is to increase the psychology-of-injury knowledge and skill usage in SMPs. An understanding of the level of individual motivation has been found to be an important component of educational intervention [6,38]. Social support during injury rehabilitation has powerful effects on athletes’ self-efficacy, anxiety level, compliance, belief in the rehabilitation process, and perceived susceptibility to reinjury. The structure of the educational intervention program included a combination of lectures, active student participation, and student interaction activities. Seminars in an observational study allowed the students to consult with an instructor, share their experiences implementing techniques with athletes, learn from others’ experiences, and receive feedback on how to address the challenges that they were facing in their work with injured athletes [6]. Each seminar began with a review of the course material and students describing the ways in which they had implemented techniques over the past week.

### 4.2. Behavioral Health Interventions in Psychology of Injury

Research has found benefits of interventions aimed at reducing mental health symptoms following sports injury, including psychoeducation, goal setting, and skills training such as visualization and imagery exercises, positive self-talk, as well as mindfulness and breathing practices. Third wave cognitive behavior therapy (CBT) interventions, such as mindfulness-based stress reduction (MBSR) and acceptance and commitment therapy (ACT), have also been found to be useful during the injury-rehabilitation process, primarily for the adult population [1]. Despite the lack of robust evidence in pediatric populations, such interventions are important to consider when working with young athletes. In addition to implementing interventions for recovery and prevention, research studies have found that injured athletes who utilize a social support system can cope more efficiently with the demands and process of injury rehabilitation than injured athletes who do not engage with social support [36].

#### 4.2.1. Psychoeducation

High-intensity training requires a high carbohydrate intake, and going into training already being dehydrated will not result in good performance [39]. There is an emphasis on the need to adapt to the training requirements and nutrition. It is imperative to educate youth and their caregivers on various aspects of sports-related injuries and recovery, career transitions, and discontinuation. Given its positive effects on athletic recovery and performance, one research study found that healthcare and sports professionals, coaches, and athletes should understand the significance of establishing psychological resilience during the injury rehabilitation process [29]. Narrative reviews highlighted the importance of integrating other healthcare professionals into the role of education on daily nutrition and body composition issues [39]. Following education, it is important to have the youth set goals for themselves using a SMART framework, with goals that are specific, measurable, achievable, relevant, and time-bound. They can then adjust their goals based on their rate of progress during the injury rehabilitation process and gain a sense of accomplishment compared to when they do not adjust their goals. Successful goal-setting interventions include setting goals that provide the youth with structure, steps, and motivation for achieving their milestones in rehabilitation [40]. Goal-setting must also be collaborative and specific to individual needs.

#### 4.2.2. Skills-Training

Youth development varies widely, and sports-related injuries and their impact on youth also vary. Teaching youths to adapt psychological skills training to the specific needs of developing athletes is an important consideration in developing skills training. Skills should be individualized to one’s daily routine and focus on long-term development rather than the short-term success of athletes. Additionally, a global skills program that includes teaching self-, volitional, motivational, and recovery skills should be an aim of training [39]. Teaching youth visualization and imagery exercises, positive self-talk, mindfulness, and breathing practices can improve their mental well-being during the recovery process, which in turn can improve their overall performance outcomes [29,40]. The regular practice of adequate warm-up routines, strength and conditioning programs, and injury prevention strategies, such as practicing good forms and techniques, can also help minimize the risk of future injuries and contribute to long-term performance sustainability [29].

While CBT has been found to be a sound intervention for injury rehabilitation, third-wave therapies such as MBSR and ACT have been researched more in adults than in youth [1]. However, given the evidence base, they are worth considering for the youth athletic population. MBSR has been found to be effective in individuals dealing with stress, pain, and symptoms of anxiety and depression. The intervention comprises activities including body scans, gentle yoga, and sitting meditation that are designed to help individuals identify their own behavioral patterns, respond thoughtfully to stressful situations by managing negative emotions, and increase pain tolerance [1]. These skills are thought to help facilitate injury recovery and improve the mental health of injured athletes. ACT focuses on accepting difficult emotions and experiences as well as changing maladaptive responses or behaviors to ultimately improve psychological flexibility [1]. In improving psychological flexibility, individuals are able to be present and have awareness of their body and mind, which can then assist in the injury-rehabilitation process as well as improve mental health symptoms.

#### 4.2.3. Maintenance

There exist three distinct theoretical perspectives related to sports injury and social support, all focusing on a perspective—the stress and coping perspective, the social constructionist perspective, and the relationship perspective [22]. Creating a social environment to enhance social support is another aspect of adapting training to better support one’s needs. Social support systems from various environments, such as parents, coaches, teammates, physical therapists, and surgeons, have been suggested to maximize recovery and maintenance [2]. A social environment is characterized by the implementation of open communication and cohesion within the training group [6,39]. Supporting young athletes in balancing their sports, education, family, and personal life is also an aspect of creating a social and positive environment for them.

A proper diet with adequate consumption of macronutrients such as carbohydrates, proteins, and fats, and micronutrients such as vitamins and minerals, as well as hydration, is essential for promoting recovery and optimizing performance in the youth’s body and athletic development [29]. Monitoring athletes’ mental health may also help identify athletes at risk of injuries and insufficient recovery.

## 5. Conclusions

Sports injuries are a common cause of mental health concerns in children and adolescent athletes. Approximately 60 million youths participate in sports or recreational activities in the United States. While sports participation is positively correlated with physical and mental health benefits, it can also result in approximately 2–3.8 million injuries each year in the United States, ranging from musculoskeletal injuries to sport-related or recreational TBIs. Given the complexity of each youth athlete’s perceived experience of injury and psychosocial responses to injury, help-seeking behaviors vary. Their perception of the role of the SMP during the rehabilitation process is crucial to the success of their recovery.

A multidisciplinary team-oriented approach has been found to be the most effective in implementing rehabilitation strategies for youth athlete recovery. Thus, multidisciplinary approaches serve a crucial role in the integration of mental health specialists into pediatric sports medicine teams to maximize sports health outcomes and the recovery process for children and adolescents. Interventions during the recovery and prevention process should include psychoeducation on sports injuries, collaborative goal setting, individualized skills training, and ongoing assessment and maintenance. Injuries are an inevitable negative consequence of participation in sports or recreational activities, and attention is paid to the developmental aspects, psychosocial factors, and treatment interventions that can help minimize the developmental, physical, and psychosocial consequences of sports-related injuries in young athletes.

### Research Gaps

As discussed, existing literature brings attention to the predisposing and risk factors, developmental aspects, and psychosocial and stress factors related to sports-related injuries for children and adolescents. However, much of the research on psychological resilience, motivation levels, and behavioral health interventions for recovery from injury is limited in pediatric literature. Future studies should consider exploring the longitudinal effects of these factors and interventions for children and adolescents. Gender and sport differences, given their complexity and that they are constantly changing, should also be explored to fill the research gaps. As medical and behavioral healthcare continue to adapt to the modern world of technological innovations and advancements, the development of digital interventions for recovery should also be considered to increase accessibility and maximize health outcomes.

## Data Availability

No new data were created or analyzed in this study. Data sharing is not applicable to this article.

## Abbreviations

The following abbreviations are used in this manuscript:

HPA	Hypothalamic–pituitary–adrenal
AT	Athletic trainer
PTSD	Post-traumatic stress disorder
ACL	Anterior cruciate ligament
SMP	Sports medicine professional
ATS	Athletic training students
CBT	Cognitive behavior therapy
MBSR	Mindfulness-based stress reduction
ACT	Acceptance and commitment therapy
